# Smart control of CAR-T cells: emerging strategies for safer and more effective cancer immunotherapy

**DOI:** 10.3389/fimmu.2026.1746673

**Published:** 2026-03-10

**Authors:** Guo-kai Zhang, Hai-mei Wang, Fang Zhou

**Affiliations:** 1 Department of Hematology, The 960th Hospital of The People’s Liberation Army (PLA), Joint Logistics Support Force, Jinan, Shandong, China; 2 First Clinical Medical College, Shandong University of Traditional Chinese Medicine, Jinan, Shandong, China; 3 Department of Oncology, Zibo Central Hospital, Zibo, Shandong, China

**Keywords:** antigen escape, cancer immunotherapy, CAR-T cells, cytokine release syndrome, logic gating, safety switches, smart control, tumor microenvironment

## Abstract

Chimeric Antigen Receptor (CAR)-T cell therapy has developed cancer immunotherapy but remains restricted by severe toxicities, antigen escape, and loss of efficacy in solid tumors. Recent advances in smart control systems aim to enhance the safety and precision of CAR-T therapies through tunable, reversible, and context-dependent mechanisms. These include the importance of inducible CAR expression, logic-gated receptors, and external control systems using drugs, light, or biomaterials. Synthetic biology approaches integrating sensor circuits and feedback loops are paving the way for programmable immunity, enabling dynamic adjustment of CAR-T activity in real time. The aim of this study is to review recent advances in strategies that enable smart controlled and designed activity of CAR-T cells for safer and more effective cancer immunotherapy. It seeks to summarize key molecular, genetic, and synthetic approaches designed to regulate CAR-T cell activation, persistence, and cytotoxicity with high precision.

## 1 Introduction

Chimeric Antigen Receptor (CAR)-T cell therapy has emerged as a transformative approach in the treatment of hematologic malignancies, demonstrating remarkable clinical efficacy in refractory leukemia and lymphoma (1). However, despite its success, several limitations continue to hinder its broader application, particularly in solid tumors (2). Uncontrolled activation, cytokine release syndrome (CRS), neurotoxicity, and antigen escape remain significant barriers. In response, current research has shifted toward engineering *smart* and *controllable* CAR-T systems that integrate synthetic biology tools to achieve tunable activation, spatial precision, and reversible safety mechanisms (3). These developments reflect a growing trend in immunotherapy: moving from static, constitutively active CAR designs toward dynamic and programmable immune responses that can be externally or intrinsically regulated (4, 5).

Recent review articles emphasize the rapid evolution of strategies for controlling CAR-T activity at transcriptional, translational, and functional levels. Key innovations include drug-inducible switches, logic-gated CAR architectures, suicide mechanisms, and environmental sensors responsive to tumor-specific cues. Collectively, these technologies aim to enhance both safety and efficacy by allowing clinicians to fine-tune immune responses in real time (6, 7). This review aims to explore and analyze current technologies designed to regulate CAR-T cell activity, focusing on light-based activation, molecular switches, biomaterial platforms, and synthetic receptor circuits. The goal is to highlight how these strategies enable precise control over CAR-T function, and to examine their mechanisms, benefits, limitations, and potential for clinical use.

## 2 Controlled CAR-T cell activity

CAR-T cell therapy has achieved remarkable success, but controlling the activity of these engineered T cells is crucial to enhance safety and efficacy. Unbridled CAR-T activity can lead to severe toxicities like cytokine release syndrome and damage to healthy tissues (on-target/off-tumor effects), especially in solid tumors (1). The clinical success of CAR-T cell therapy in hematologic cancers has validated the concept of redirecting a patient’s immune system to recognize and destroy tumor cells. Yet, the potent and often uncontrollable nature of engineered T cells can result in life-threatening toxicities and limited performance in complex tumor microenvironments. To address these challenges, next-generation CAR-T systems are being designed with built-in control mechanisms that regulate their activation, persistence, and cytotoxicity. Controlled CAR-T cell activity seeks to achieve an optimal balance between efficacy and safety, sustaining antitumor potency while minimizing collateral tissue damage and systemic inflammation (3).

One fundamental approach involves transcriptional and post-translational control. Inducible promoters and regulatory elements enable reversible CAR expression under defined conditions. Systems such as the tetracycline (Tet-On/Tet-Off) or rapamycin-dependent promoters can toggle CAR expression with small molecules. This design allows clinicians to modulate CAR-T cell activity dynamically during treatment, adjusting intensity or shutting down responses in case of toxicity. Similarly, post-translational switches based on proteolytic cleavage or drug-inducible dimerization can fine-tune CAR signaling at the protein level, providing rapid and reversible control without permanently altering T cell viability (7).

### 2.1 Light-based activation

**Figure 1:**
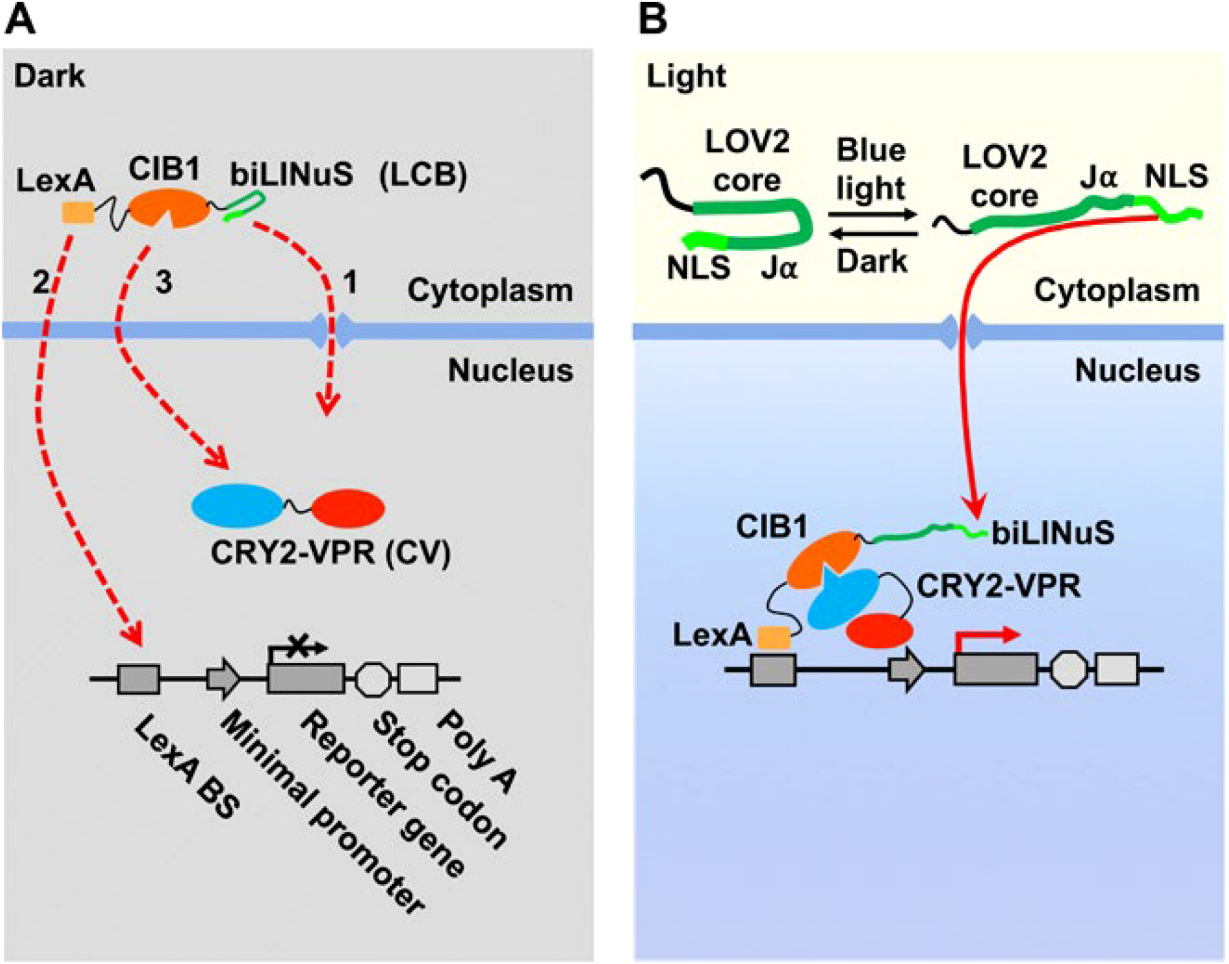
Overview of the LINTAD gene activation system. **(A)** LINTAD consists of three elements (1): LexA-CIB1-biLINuS (LCB), combining LexA, CIB1, and a light-responsive NLS (2); CRY2PHR-VPR (CV); and (3) a light-inducible reporter with LexA binding sites and a minimal promoter. In darkness, LCB remains cytoplasmic while CV is nuclear. **(B)** Blue-light exposure unfolds the LOV2 domain in biLINuS, exposing the NLS and driving LCB nuclear import. LexA binds the reporter’s LexA sites, and CRY2PHR associates with CIB1, recruiting VPR to the promoter and activating gene expression. Reprinted with permission from “Engineering light-controllable CAR T cells for cancer immunotherapy” by Huang Z. et al., licensed under CC BY 4.0, Science Advances (2020).

Light-controlled CAR-T cells (photoactivatable-CAR-Ts) use optogenetic tools to achieve *spatiotemporal precision* in T cell activation. In these designs, CAR-T cells remain inert until a specific light signal is delivered, allowing physicians to target activation to tumor sites and time windows. For example, one strategy is to split the CAR signaling domain and attach photoreceptor modules that dimerize under light. Huang et al. engineered a CAR T with a light-inducible nuclear dimerization system (LINTAD) to control gene expression and T cell activation by blue light pulses (3). Similarly, a “LiCAR” (light-switchable CAR) was created by installing photo-responsive domains into a split CAR; only upon blue-light illumination do the two halves assemble into a functional CAR, triggering T cell cytotoxicity. This enables on-demand activation of CAR-T cells with a high degree of control (1, 8) (**Figure 1**). 

Notably, light-based CAR-T control can be made practical for deep tissues by using near-infrared (NIR) light in conjunction with specialized materials. NIR light penetrates deeper into the body, but is not directly absorbed by typical optogenetic switches (9). To bridge this gap, researchers have employed up-conversion nanoparticles (UCNPs) that convert tissue-penetrant NIR into local blue light. When LiCAR-T cells were combined with NIR-to-blue UCNP “transducers,” it enabled *wireless* activation of CAR-T cells inside living animals (10). This platform achieved spatially confined CAR-T activity and temporal control over dosing and duration of T cell responses, greatly mitigating systemic side effects (1). The major advantage of light-based control is this unparalleled precision – clinicians could, in principle, illuminate only the tumor region (e.g. via an endoscope or external beam for superficial lesions) to activate CAR-T cells *in situ*, reducing collateral damage. Additionally, the activation is quickly reversible by turning off the light. However, there are limitations (11). Light delivery to all disease sites can be challenging (especially for deep or disseminated tumors), and requires specialized hardware. Furthermore, continuous or repeated illumination might be needed to maintain CAR activity, and immune responses to any introduced light-sensitive proteins must be considered (11). Despite these hurdles, optogenetically controlled CAR-T systems demonstrate a powerful proof-of-concept for externally regulating cell therapies with exquisite precision, and ongoing advancements (such as red-shifted light sensors or implantable light sources) aim to improve clinical feasibility (12). 

### 2.2 Molecular switches (small-molecule control)

Another major approach to control CAR-T cells uses small molecules as triggers or “switches” to modulate T cell activity. These pharmacologically regulated CAR-T systems allow doctors to turn the cells on or off, or even eliminate them, by administering a specific drug (19).

#### 2.2.1 Drug-gated ON-switch CARs

**Figure 2:**
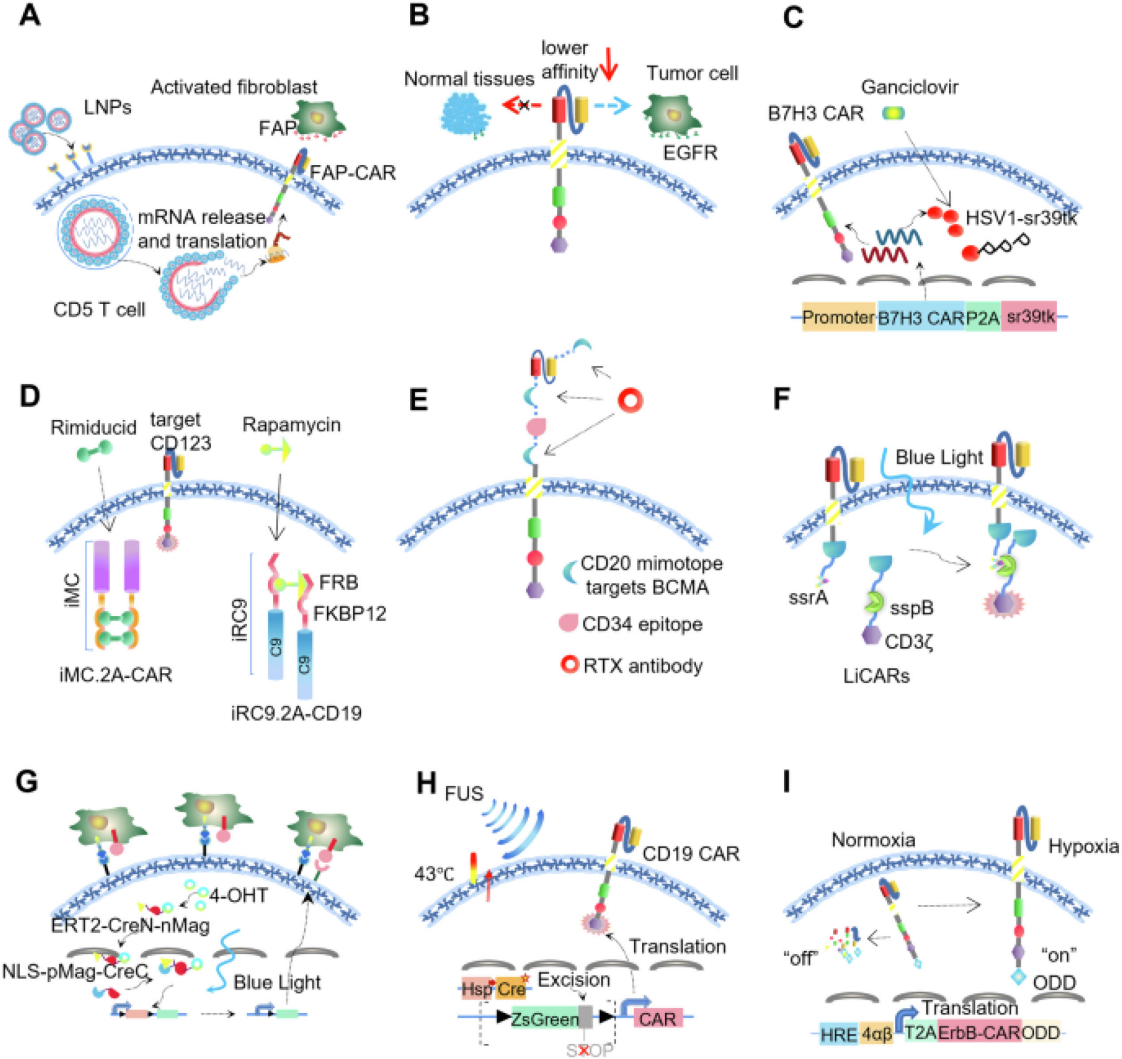
Small-molecule and molecular switch mechanisms in CAR-T regulation (1). Self-control systems – regulating CAR expression (CD5/LNP-FAP CAR-T, **A**) or binding affinity (EGFR CAR-T, **B**) (2). Switch-off systems – introducing suicide mechanisms, including TK-ganciclovir (B7H3-sr39tk, **C**), iCasp9 (iRC9, **D**), or RTX-CD20 (CubiCAR-T, **E**) (3). Switch-on systems – activating CAR-T cells by external stimuli such as light (LiCAR-T, TamPA-Cre; **F–G**), focused ultrasound (FUS-CAR-T, **H**), or hypoxia (HypoxiCAR-T, **I**). Reprinted with permission from “CAR-T therapy dilemma and innovative design strategies for next generation” by Wang Z. et al., licensed under CC BY, Cell Death & Disease (2025).

These CAR-T cells are engineered so that their activation *requires* the presence of a benign small molecule (**Figure 2**). In one seminal design, the CAR was split into two halves – one containing the antigen-binding domain and the other the signaling domains – each fused to a pair of proteins that heterodimerize only when a specific drug is present (20). For example, an “ON-switch” CAR used a rapamycin analog to bring together FKBP and FRB domains on the two CAR halves (21). In the absence of the drug, the CAR halves do not assemble, so T cells remain inactive even if they encounter antigen. When the drug is given, it induces assembly of a functional CAR, *activating* the T cells only under those conditions. This system retains antigen specificity but adds an extra layer of control, enabling physicians to precisely tune the timing and intensity of the immune response (20). The CAR-T activity becomes titratable by adjusting drug dose, and reversible by withholding the drug. A key benefit is the ability to avoid excessive T cell activity – for instance, a low dose of the dimerizer might elicit a mild cytotoxic response to start, reducing the risk of cytokine storm, whereas a higher dose could ramp up the attack if the patient is tolerating well (22). One limitation, however, is the need for continuous presence of the drug to maintain CAR assembly; the pharmacokinetics of the compound will dictate how tightly one can control the cells. Additionally, any off-target effects of the small molecule (e.g. rapamycin’s immunosuppressive properties) must be taken into account. Still, drug-gated CAR switches represent a powerful strategy for on-demand activation of cell therapies (23).

#### 2.2.2 Inducible “suicide” switches

In addition to turning CAR-T cells on, it is equally important to have an emergency “off” switch to quickly terminate the cells if severe toxicity occurs. One widely adopted solution is the inducible Caspase-9 (iCasp9) suicide switch. CAR-T cells are genetically modified to express an inert form of the Caspase-9 enzyme fused to a drug-binding domain. Upon administration of a specific small molecule (AP1903, also known as rimiducid), the drug binding domains dimerize and activate Caspase-9, triggering apoptosis in the engineered T cells (19). This results in rapid self-destruction of the CAR-T population, ideally halting any life-threatening immune reactions. Notably, the iCasp9 system has been tested in early clinical trials: in a phase I setting, patients receiving CAR-T cells with iCasp9 could be treated with the drug if severe side effects emerged, leading to elimination of the transferred cells within minutes (25). An interesting observation is that activated CAR-T cells (which often express higher levels of the transgene) may be preferentially killed, potentially quelling the most dangerous cells first. The clear advantage of a suicide switch is the safety net it provides. It addresses a major concern of gene therapies by allowing the therapy to be aborted after deployment. The downside is that it’s a one-time, irrevocable off-switch once triggered, the therapeutic benefit is lost along with the cells. Therefore, it would only be used in extremis. Ongoing efforts aim to refine such safety switches (for example, using lower doses of dimerizer to partially deplete cells, or designing reversible kill switches), but iCasp9 remains a leading safeguard in the CAR-T toolbox and has been incorporated into several next-generation CAR designs in clinical development (25).

#### 2.2.3 Reversible signaling inhibitors

Rather than genetic switches, researchers have also discovered that certain existing drugs can act as *temporary off-switches* for CAR-T cells. A prime example is the tyrosine kinase inhibitor dasatinib, a leukemia drug that was found to *pause* CAR-T cell activation in a reversible manner (26). Dasatinib blocks proximal T-cell signaling (LCK kinase activity), thereby halting CAR T cells’ cytolytic function, cytokine release, and proliferation within hours of administration. Importantly, when dasatinib is cleared or withdrawn, CAR-T cells can resume their activity, essentially functioning as a “remote control brake” on the therapy (27). Clinically, this approach could be used to manage acute toxicities – for instance, a patient showing early signs of cytokine release syndrome could receive a dose of dasatinib to immediately dampen T cell activity and prevent escalation, and once the patient is stable, the drug is stopped to let the CAR-T cells continue their work. The benefit here is fine temporal control using an already-approved drug. The limitation is that global T cell inhibition might also reduce anti-tumor efficacy during the period of inhibition, so timing and dosing are critical. Nonetheless, this strategy exemplifies how pharmacologic agents can be repurposed to create an *on/off switch* for cell therapies without additional genetic engineering (28).

#### 2.2.4 Modular adapter systems

Another molecular control strategy involves decoupling antigen recognition from T cell activation using adapter molecules. So-called “universal” CAR-T platforms have been designed where the CAR on the T cell recognizes a universal tag or ligand (instead of a tumor antigen directly), and a separate *adapter* (usually a bispecific antibody or conjugate) bridges the CAR to the tumor cell. One early example is a CAR that binds biotin; T cells were redirected to tumor by administering biotinylated antibodies against the tumor antigen (29). More recently, a two-component system called zipCAR was created: T cells express a CAR with an inert extracellular leucine zipper, and an accompanying soluble “zipFv” adapter is a tumor-specific scFv fused to the matching zipper. By administering different adapters, the same CAR-T cells can be retargeted to various antigens, or tuned by varying adapter dose (30). This acts as a molecular *switch* without the adapter, CAR-T cells ignore target cells; with the adapter, they form a complex and attack. The ability to dynamically redirect or modulate CAR-T specificity is highly useful for cancers that evolve new antigens or for treating multi-focal disease. It also adds a safety control – stopping adapter infusion should render the T cells inert, much like taking away the key. The challenges include the need for repeated dosing of protein adapters (which could provoke immune responses or have their own kinetics) and ensuring the adapter does not itself cause off-target effects. Despite these issues, adapter-mediated CAR-T cell control is a promising avenue, and early trials (e.g. using anti-FITC CARs with FITC-labeled antibodies, or the universal “UniCAR” system) are exploring the feasibility of this pharmacologically *programmed* cell therapy (8, 30).

#### 2.2.5 Vaccine-boosted CAR-T therapy

**Figure 3:**
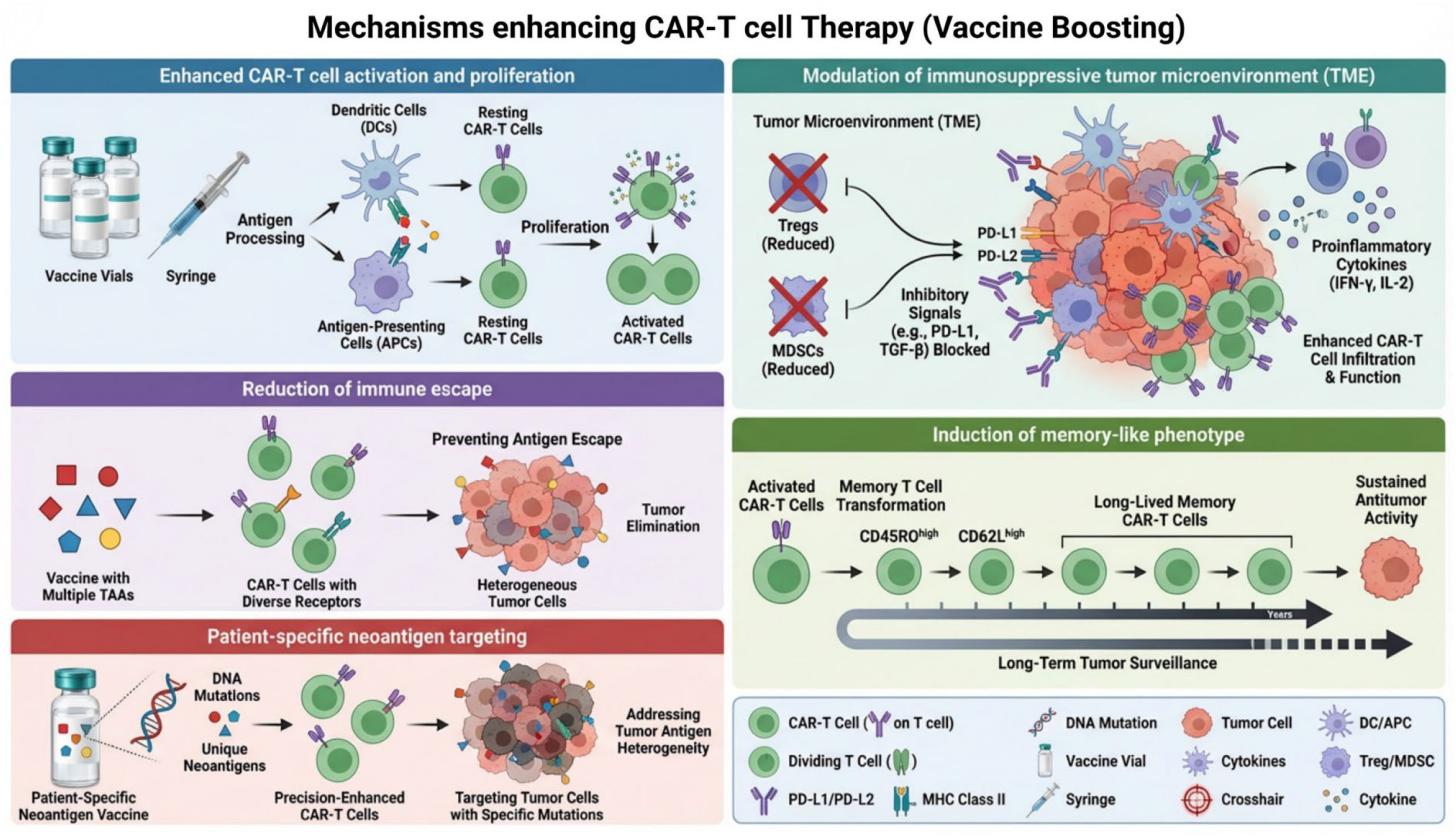
Synergistic mechanisms of vaccine-boosted CAR-T cell therapy. Vaccines enhance CAR-T cell efficacy through multiple complementary pathways: Enhanced Activation & Proliferation: Vaccine-primed dendritic cells present tumor antigens, delivering strong activation and co-stimulatory signals to CAR-T cells, driving robust expansion and effector function. TME Reprogramming: Vaccines reduce immunosuppressive cells (Tregs, MDSCs), block inhibitory checkpoints (e.g., PD-1), and promote pro-inflammatory cytokines, creating a permissive environment for CAR-T cell infiltration and activity. Memory Formation: Vaccine stimulation promotes differentiation of CAR-T cells into long-lived memory subsets (e.g., central memory T cells), enabling sustained antitumor surveillance and recall responses. Prevention of Immune Escape: By presenting a broad antigen repertoire, vaccines work with multi-targeting CAR-T cells to counteract tumor antigen loss, allowing recognition through multiple independent pathways (CARs, TCRs). Personalized Neoantigen Targeting: Patient-specific neoantigen vaccines elicit responses against unique tumor mutations, addressing heterogeneity and complementing the specificity of the CAR construct. Together, these mechanisms overcome key limitations of CAR-T monotherapy, including poor persistence, immunosuppression, and antigen escape.

Beyond the direct pharmacologic control of CAR-T cells, an emerging “systems-level” strategy seeks to amplify and sustain antitumor immunity by productively engaging the endogenous immune system. Termed vaccine-boosted CAR-T therapy, this approach employs bispecific engagers or molecular chimeras designed to physically bridge CAR-T cells with antigen-presenting dendritic cells (DCs) (31, 32). For instance, a bispecific antibody targeting a CAR-engineered domain (e.g., a peptide tag on the CAR) and a DC-specific receptor (e.g., CD40) can co-localize the two cell types (33). This forced interaction triggers robust DC activation and maturation, leading to the processing and presentation of tumor-derived antigens beyond the initial CAR target, a process known as epitope spreading (33). The key benefit of this strategy is its ability to overcome two major limitations of conventional CAR-T therapy: antigen escape and poor long-term persistence. By initiating a broad, endogenous T-cell response alongside the direct cytotoxicity of the engineered cells, it creates a self-amplifying, systemic immune attack that can control heterogeneous tumors and provide durable immunological memory (34). Seminal studies have demonstrated the potency of this concept using various molecular designs, including small-molecule-based adapters (35), Fc-engineered bispecific antibodies (36), and more complex chimeric fusion proteins (37). By programming critical interactions within the tumor microenvironment, vaccine-boosted CAR-T therapy represents a sophisticated form of smart control that shifts the therapeutic paradigm from a solitary “living drug” to a coordinated, *in situ* cancer vaccination event (**Figure 3**).

Several distinct vaccine modalities have demonstrated synergistic potential in preclinical models to enhance CAR-T cell expansion, functionality, and antitumor efficacy. mRNA vaccines formulated with lipid nanoparticles, such as CLDN6-encoding RNA-lipoplexes, efficiently transfect DCs in lymphoid organs, enabling systemic antigen presentation that significantly amplifies CAR-T cell engraftment, polyfunctionality, and tumor regression even at subtherapeutic CAR-T doses (38, 39). Peptide-based vaccines, particularly when engineered as amphiphilic ligands (e.g., EGFRvIII-polymer conjugates), overcome rapid degradation by binding albumin and trafficking to draining lymph nodes, where they decorate APC surfaces to provide direct CAR-mediated stimulation and promote broad endogenous T-cell responses against heterogeneous tumors (40, 41). Viral vector vaccines, including recombinant vaccinia viruses encoding tumor antigens (e.g., gp100) and oncolytic viruses engineered to express CAR targets (e.g., truncated CD19), leverage their inherent tropism to deliver antigens directly to tumor sites or APCs, thereby activating CAR-T cells via their native TCRs and enhancing local immune infiltration and durable remission (42). DC-based vaccines, involving ex vivo antigen-loaded autologous DCs (e.g., WT1-pulsed DCs), exploit the potent antigen-presenting capacity of DCs to prime and restimulate CAR-T cells specifically, leading to increased tumor infiltration and enhanced cytotoxicity, though challenges remain regarding their persistence and migration *in vivo* (43). Collectively, these vaccine strategies represent complementary approaches to overcome CAR-T cell limitations by orchestrating.

### 2.3 Biomaterial-based control of CAR-T cells

Bioengineering approaches using biomaterials offer another dimension of control for CAR-T cell therapy. Instead of (or in addition to) genetic modifications, biomaterial strategies focus on *where* and how CAR-T cells are delivered and activated in the body by creating supportive niches or physical constraints (44). These methods can localize CAR-T activity to tumors and improve T cell functions through engineered microenvironments:

#### 2.3.1 Injectable CAR-T cell niches

One innovative strategy is to co-deliver CAR-T cells with a supportive scaffold or hydrogel that localizes them at the tumor site and provides sustained stimulatory signals. Grosskopf et al. developed a transient injectable hydrogel that serves as a local immune niche for CAR-T cells The hydrogel is formulated to encapsulate CAR-T cells along with cytokines (such as IL-15 and other factors) and is injected directly into or next to the tumor. This biomaterial scaffold has a porous structure that *permits T-cell migration* but *retains critical cytokines*, preventing them from diffusing away (45). The result is a concentrated microenvironment that keeps the CAR-T cells in the tumor vicinity and promotes their proliferation and function *in situ*. In mouse models of solid tumors, delivering CAR-T cells in such a stimulatory hydrogel markedly enhanced T-cell expansion at the tumor and improved anti-tumor efficacy, compared to traditional systemic infusion (46). The hydrogel gradually degrades over time, so it provides a transient but potent boost, effectively acting as a local “charging station” for CAR-T cells. The benefits of this approach include better CAR-T cell persistence in the hostile tumor microenvironment and reduced systemic exposure (since the cells are largely kept at the target site). Clinically, this could translate to higher tumor kill with fewer side effects. The limitations involve the need for a local injection (an interventional procedure) and the challenge of treating metastatic disease confined to one or few sites. Nonetheless, this concept of locoregional delivery using biomaterials is a promising way to control CAR-T distribution and functional state after administration (47).

#### 2.3.2 Implantable bioreactors for CAR-T manufacture

An exciting extension of the biomaterial approach is to actually generate CAR-T cells *inside the patient* at the desired location. Agarwalla et al. introduced an implantable alginate scaffold (MASTER) that acts as an *in vivo CAR-T factory* (48). In this method, a scaffold loaded with patients’ native T cells (not yet CAR-modified) and viral vectors encoding a CAR is surgically placed at a site (e.g. subcutaneously). The porous alginate scaffold facilitates viral transduction of T cells on-site and their expansion. Over the course of a few days, functional CAR-T cells are produced *in vivo* and released from the scaffold into the circulation (48, 49). In a preclinical study, the MASTER scaffold seeded with human T cells and CAR virus successfully generated CAR-T cells in a mouse, which then trafficked to tumors and cleared them (50). Remarkably, the *in vivo*-produced CAR-T cells showed greater persistence and potency than conventionally manufactured cells. This biomaterial-based *in situ* manufacturing offers several potential advantages: it obviates the lengthy ex vivo manufacturing process (reducing vein-to-vein time to essentially one day), and it localizes initial T-cell activation/expansion to a specific site, which might enhance safety (since the potent activation occurs in a controlled implant, not systemically) (51). Additionally, these scaffolds can be laden with supporting factors like cytokines or antigen-presenting cells as needed, tailoring the niche for optimal T cell programming (52). The challenges ahead include ensuring consistent and complete CAR gene transfer *in vivo* and managing the immune response to the scaffold or viral vector. This approach is at an early stage, but it represents a convergence of cell therapy and tissue engineering to control the production and deployment of CAR-T cells within the body (53).

#### 2.3.3 Other biomaterial innovations

Beyond scaffolds and hydrogels, researchers are exploring various biomaterial tools to control CAR-T cells. For instance, nanoparticle delivery systems can concentrate CAR-T cells or requisite signals in certain tissues. One example is using magnetic nanoparticles that guide T cells to tumor sites under an external magnetic field, or nanoparticles that release chemoattractant to draw T cells into a tumor. Additionally, localized drug depots (e.g. a gel that slowly releases a CAR-T attracting chemokine or an activating drug at the tumor) can modulate where CAR-T cells go and become active (50). These strategies seek to confine the immune attack to the tumor microenvironment and shield normal tissues. The field of biomaterials for immunotherapy is rapidly expanding, and CAR-T cell therapy stands to benefit from devices that provide spatial and temporal control, essentially *macro-scale* regulation to complement the genetic and molecular controls at the cell scale. While any implanted material or device introduces additional considerations (biocompatibility, retrievability, potential infection risk), the payoff could be safer and more effective therapies, especially for solid tumors that have been less responsive to free-roaming CAR-T cells (54).

### 2.4 Synthetic receptors and logic-gated CAR-T cells

A highly innovative avenue for control is the design of synthetic receptors that imbue T cells with Boolean logic capabilities, essentially programming the cells to make more complex decisions before killing a target. Traditional CARs recognize a single antigen and trigger activation immediately. Synthetic receptor circuits can require multiple conditions (antigens) to be met, or can veto activation in certain contexts, thereby adding *logical control* over CAR-T cell responses (52) (**Table 1**).

**Table 1:** Light-based control systems for CAR-T cell activation.

Control platform/system	Light wavelength	Mechanism	Primary advantage	Translational challenge	References
LiCAR	Blue light (≈480 nm)	Photo-induced dimerization of split CAR halves into a functional receptor	Rapid, reversible *ON* switch with precise spatial activation	Limited tissue penetration of blue light (sub-millimeter)	(1, 10)
LINTAD	Blue light (≈460 nm)	Light-inducible nuclear translocation and dimerization system driving CAR gene expression (LexA–CRY2/CIB1-based).	Real-time, tunable transcriptional *ON* switch for CAR in T cells (tested *in vitro*/*in vivo*).	Requires continuous illumination hardware; blue light penetration is shallow	(11)
UCNP-LiCAR	NIR (≈980 nm) upconverted to blue	Upconversion nanoparticles (UCNPs) convert deep-penetrating NIR into CAR-activating blue light	*Non-invasive in vivo* activation of LiCAR-T cells with centimeter-range penetration	Added complexity: injectable nano-transducers and surgical placement/removal of particles	(13)
Dual-Input CAR (AND gate)	Blue light + drug (Tamoxifen)	Two-step control: Tamoxifen primes an engineered CAR-T, then blue light triggers nuclear import/Cre-lox recombination to express CAR	Extremely low “accidental” activation – requires both inputs; spatiotemporal *AND* logic for safety	Requires systemic drug administration and localized illumination; blue light depth limits usage to superficial tumors	(12)
Photothermal CAR	NIR laser (≈808 nm)	NIR absorbed by gold nanorods generates mild heat (40–42 °C) to trigger a heat-inducible HSP promoter driving CAR expression	*Remote in vivo* CAR activation with deeper tissue reach (NIR penetrates > mm); ~20-fold induction of gene expression in T cells	Requires injecting exogenous plasmonic nanoparticles as transducers; thermal dose must be finely controlled to avoid damage.	(9)
Photo switchable Adaptor	UV (365 nm)	A bifunctional small-molecule adaptor (e.g. folate–fluorescein) bridges CAR to tumor antigen; UV light cleaves a linker to disrupt this “chemical CAR” bridge	*On-demand OFF switch*: external light can terminate CAR-T activity to prevent toxicity; provides reversible and spatial control over CAR targeting	Relies on UV light (poor penetration, potential phototoxicity) and continuous administration of the adaptor molecule.	(14, 15)
Photocaged Adaptor	UV (365–405 nm)	Tumor-targeting antibody is tagged with a fluorescein derivative “caged” by a photolabile group; UV light uncages fluorescein, enabling its recognition by a FITC-specific CAR	*Precision targeting in* sp*ace and time*: CAR-T cells can be activated only at illuminated tumor sites	Shallow light penetration necessitates special delivery devices; potential immunogenicity of the FITC tag on the adaptor	(16)
Melanopsin Opto-cytokine T cells	Blue light (≈450 nm)	Human melanopsin (GPCR) is expressed in T cells; light stimulation triggers G_q_/PLC signaling and NFAT-driven expression of cytokines (IL-2, IL-15, TNF-α)	Enhances expansion and cytotoxicity of CAR-T in solid tumors by on-demand cytokine release	Blue light penetration is low (few mm); requires vitamin A-derived cofactor (endogenous retinal) for melanopsin activity.	(17, 18)

#### 2.4.1 AND-gate dual CAR systems

To improve specificity, researchers have created CAR-T cells that need to sense *two different antigens* on a target cell to fully activate. One implementation is the dual CAR or “split signaling” approach: the T cell is engineered with two CARs – one provides signal 1 (e.g. the CD3ζ activation domain) upon binding antigen A, and the other provides signal 2 (co-stimulation, e.g. CD28 or 4-1BB domain) upon binding antigen B (55, 56). Individually, neither CAR can trigger full T cell activation, but when a cell such as a tumor expresses both A and B, the two signals combine to fire the T cell. This acts as an AND gate requiring co-expression of two antigens. For example, to distinguish tumors from normal cells, one antigen might be a tumor-associated marker and the second an organ-specific marker – only the overlap (presumably unique to tumor tissue) leads to T cell killing. Preclinical studies (such as those targeting PSMA and PSMA’s prostate-specific partner in prostate cancer) have shown that dual CAR T cells can spare single-antigen cells while attacking double-positive tumor cells. The benefit of an AND gate is a higher discriminatory power, potentially reducing attack on healthy cells that might express one of the antigens at low levels (57). The trade-off is that if the tumor does not uniformly express both markers, there is a risk of escape (cells expressing only one antigen would be missed). Thus, careful selection of antigen pairs is essential. Dual CAR designs have moved into clinical testing for certain solid tumors, as they promise to widen the therapeutic window by increasing specificity at the cost of requiring dual antigen presence (58).

#### 2.4.2 Inhibitory CARs (NOT gates)

Rather than requiring two positives, another strategy is to include a receptor that actively suppresses T cell activation when it encounters a forbidden antigen. These are known as inhibitory CARs, or iCARs, functioning as a logical NOT gate (52). An iCAR typically consists of an extracellular scFv that recognizes an antigen expressed on healthy tissue (but not on the tumor), fused to an intracellular checkpoint signaling domain (such as the CTLA-4 or PD-1 cytoplasmic tail that contains immunoreceptor tyrosine-based inhibitory motifs, ITIMs). If the CAR-T cell encounters a cell displaying this normal antigen, the iCAR delivers a dominant negative signal that overrides the activation signal. In effect, the T cell is programmed to *cancel its attack* in the presence of that antigen (58). Fedorov et al. first demonstrated this concept by engineering T cells that would kill tumor cells unless they saw an antigen like HER2 (modeling a healthy tissue antigen), in which case the inhibitory signal blocked the response. iCARs thus add a layer of *auto-regulation* to avoid on-target/off-tumor toxicity. The challenge is that the inhibitory signal needs to be potent and fast enough to abort activation; if a T cell encounters both antigens simultaneously, there is a race between activation and inhibition. Studies have shown that properly tuned iCARs can indeed protect normal cells in co-culture experiments, but *in vivo* timing and antigen distribution matter (59, 60). Another limitation is that this approach relies on knowing a specific antigen that cleanly delineates healthy tissue, which may not exist for all tumor targets. Nonetheless, iCARs are a powerful concept for creating “sense-and-block” circuits in CAR-T cells. They exemplify how synthetic receptors can enforce more stringent decision-making, potentially allowing *safer targeting* of antigens that are not completely tumor-specific (61).

#### 2.4.3 Sequential AND logic with synNotch

A breakthrough in synthetic biology was the development of synthetic Notch (synNotch) receptors that enable *cascaded* antigen sensing. A synNotch receptor is a custom-built receptor where, upon binding antigen A, the receptor’s intracellular domain is cleaved and releases a transcription factor that activates expression of a chosen gene (for example, a CAR targeting antigen B) (62). This creates a programmed sequence: first the T cell must encounter antigen A – this alone does *not* trigger immediate killing, but it primes the T cell by inducing it to express a CAR (or cytokine, or any gene) as a second step. Then, if the T cell later encounters a cell with antigen B, the newly expressed CAR-B will trigger full activation and cytotoxicity (62, 63). The net effect is a requirement that antigen A precede antigen B exposure, enforcing a temporal AND gate (sometimes called a “serial” or IF-THEN logic gate). For example, one can require that a T cell first receives a signal from a tumor microenvironment marker (say, a stromal or extracellular matrix protein A present only in tumor tissue) before it will deploy a CAR against tumor antigen B (63). This way, if the CAR-T is in a healthy tissue (lacking A), it never even expresses the effector CAR, and thus won’t react to B on normal cells. Roybal et al. showed that synNotch circuits can allow T cells to discriminate cancer cells in a mixed environment: only in the presence of the first antigen do they “arm” themselves to attack the second antigen-bearing target (61). The benefit of synNotch circuits is high specificity and flexibility – virtually any extracellular antigen can be used to trigger any genetic program, not just CAR expression. This opens the door to multi-antigen targeting in a highly modular way (e.g., requiring 2, 3, or even a sequence of signals to authorize a kill). Additionally, because synNotch can drive expression of not only CARs but also cytokines or co-stimulatory ligands, it offers a platform for *programmable cell therapies* that execute complex behaviors only in defined conditions (63). One limitation observed is that if tumor and normal cells are intermingled, a synNotch T cell might get “primed” by a tumor cell with antigen A and then immediately kill a neighboring normal cell with antigen B (since once the CAR-B is expressed, it will attack any B-expressing cell) (61). This is a spatial problem, requiring that A and B antigens be on the same cell is ideal, but synNotch in its basic form only imposes a sequential requirement, not necessarily co-location. Researchers are addressing this by tuning synNotch sensitivity and using additional gating layers. Overall, synNotch represents a revolutionary leap in T-cell engineering, demonstrating how custom receptors can function as *molecular logic gates* to precisely control CAR-T cell targeting and mitigate off-tumor effect (63).

#### 2.4.5 Complex logic and programmable circuits

**Figure 4:**
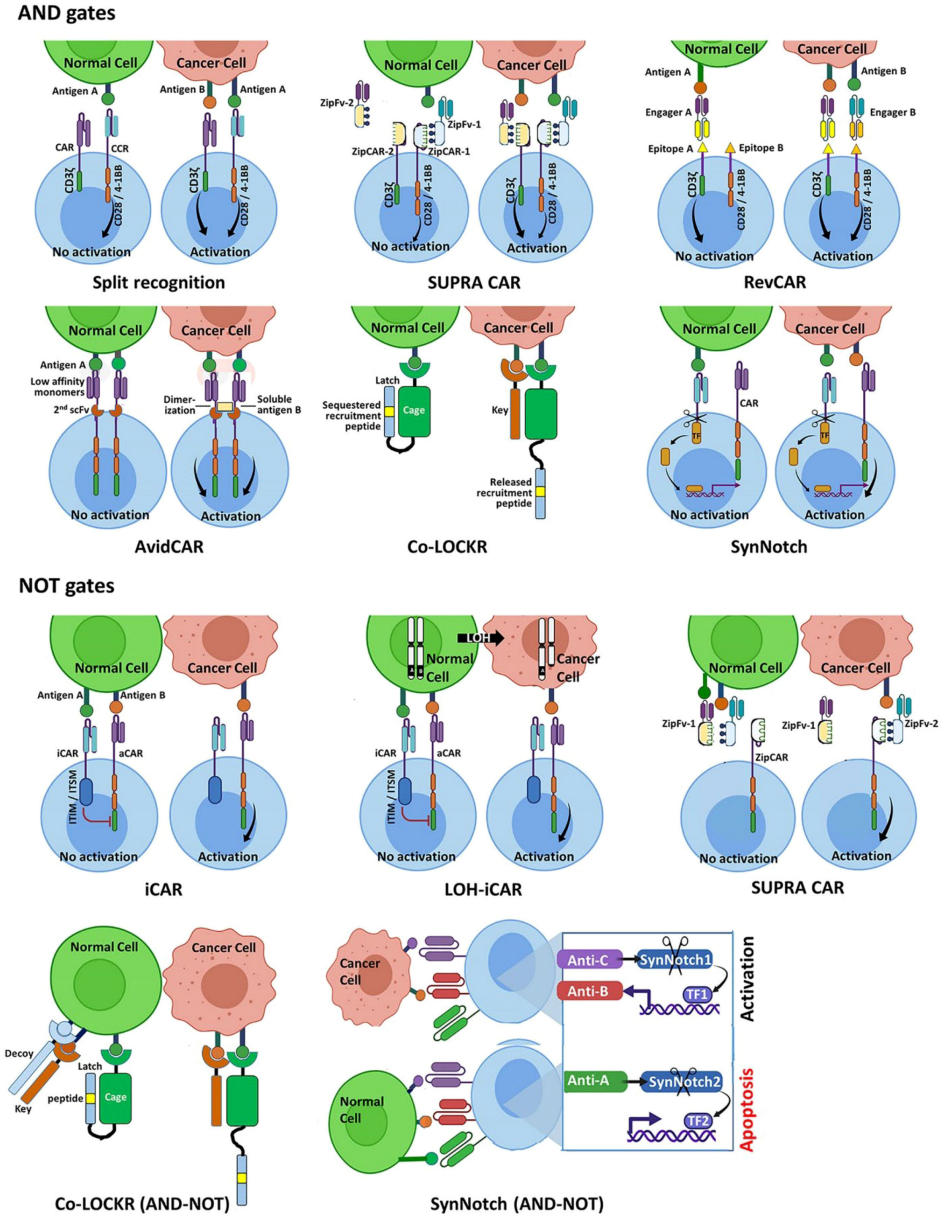
Simplified schematic of Boolean logic AND and NOT gate designs improving antigen-specific control in CAR-T therapy. AND gates: Split-receptor CARs divide signaling domains between two receptors recognizing distinct antigens; full activation occurs only when both are engaged. The SUPRA CAR system employs a universal *zipCAR* with a leucine zipper that binds soluble antigen-specific *zipFv* adaptors, allowing flexible targeting. In this example, *zipCAR-1/zipFv-1* detect antigen A (shared) and *zipCAR-2/zipFv-2* detect antigen B (tumor-specific); activation requires both. RevCARs replace scFvs with short peptide epitopes and use bi-specific engagers connecting peptide and tumor antigens; only simultaneous engagement triggers activation. AvidCARs activate through receptor dimerization induced by dual-antigen binding. Co-LOCKR uses a “Latch”–”Cage”–”Key” design: the CAR recognizes a hidden recruitment peptide that becomes exposed only when a second tumor antigen binds the Key. synNotch AND circuits release a synthetic transcription factor upon antigen A binding, inducing CAR expression for antigen B NOT gates: iCARs and LOH-iCARs use inhibitory receptor domains; binding to inhibitory antigens blocks activation despite activating signals. The SUPRA CAR NOT circuit relies on leucine-zipper competition—dual antigen engagement on normal cells locks zippers together, preventing activation, whereas tumor cells lacking one antigen allow signaling. Co-LOCKR NOT integrates AND/NOT logic using a Decoy protein that captures the Key on normal cells, stopping activation. synNotch NOT introduces a suicide module triggered by inhibitory antigens to eliminate CAR-T cells encountering normal tissues. Reprinted with permission from “Implementing logic gates for safer immunotherapy of cancer” by Savanur M.A., Weinstein-Marom H., and Gross G., licensed under CC BY, Frontiers in Immunology (2021).

Building on AND, NOT, and sequential gates, scientists are crafting even more sophisticated synthetic circuits for T cells (**Figure 4**). For instance, combinatorial antigen recognition can be extended to multi-input boolean logic (beyond just two signals). One advanced example is the Co-LOCKR system, a modular protein logic gate that can perform multi-antigen AND + NOT operations. In Co-LOCKR, two separate proteins (Cage and Key) are engineered such that they only activate a T cell when both bind to their respective antigens on the same cell, *unless* a third “Decoy” signal is present to inhibit the interaction (60). In a proof-of-concept, Co-LOCKR was used to require the presence of two tumor antigens *and* the absence of a normal antigen to trigger T cell killing (61, 64). This kind of multilayer logic is akin to adding IF-AND-NOT conditions that more precisely define target cells. It highlights the potential of programmable cell therapies: T cells can be equipped with circuits that integrate *multiple* disease signals and only respond when the exact combination is detected. The benefit, of course, is an unprecedented level of control and precision – theoretically, one could target tumors that have a unique fingerprint of 3–4 markers while ignoring any cell that doesn’t perfectly match that profile. The downsides are increased complexity and the need for larger genetic payloads (more components increase the chances of immunogenicity or malfunctions) (64, 65). Many of these complex logic-gated systems are still in preclinical development, but they represent the *future direction* of controlled CAR-T therapy. As our ability to program cells improves, we may see “smart” CAR-T cells that behave like tiny computers, executing treatment programs with minimal supervision once injected (39, 61).

## 3 Clinical translation of smart CAR-T therapies

The promising safety and efficacy profiles of smart CAR-T systems in preclinical models have catalyzed their transition into early-phase clinical trials (67). These trials aim to validate the enhanced specificity and controllability of logic-gated, switchable, and microenvironment-responsive CAR-T cells in patients, particularly for solid tumors where conventional CAR-T therapy has faced significant challenges (49). The clinical landscape, while still emerging, highlights several pioneering platforms.

The clinical trials summarized in **Table 2** represent the vanguard of smart CAR-T translation. The early safety data from the Tmod platform (EVEREST-1) are particularly encouraging, providing the first clinical evidence that a Boolean AND-NOT logic circuit can function in patients to mitigate on-target, off-tumor toxicity (74). The initiation of the SynNotch-based E-SYNC trial for glioblastoma marks another milestone, testing a sequential AND-gate in one of the most challenging solid tumor microenvironments.

**Table 2:** Clinical trials of smart - controlled CAR-T cell therapies.

Control strategy & platform	Target/indication	Clinical trial phase & identifier	Key findings	References
**Logic-Gated (AND-NOT) CAR-T** *Tmod Platform (A2 Biotherapeutics)*	CEA+ solid tumors with HLA-A*02 Loss (e.g., colorectal, pancreatic cancer)	Phase 1/2 EVEREST-1 (NCT05736731)	Favorable safety profile reported (1 Gr 2 CRS in 10 pts). One patient with metastatic pancreatic cancer achieved a confirmed partial response. Demonstrates proof-of-concept for exploiting tumor-specific antigen loss (LOH) to protect healthy tissue.	(68)
**Logic-Gated (AND-NOT) CAR-T** *Tmod Platform (A2 Biotherapeutics)*	Mesothelin (MSLN)+ solid tumors with HLA-A*02 Loss	Phase 1/2 EVEREST-2 (NCT06051695)	Actively recruiting. Aims to evaluate safety and efficacy of this logic-gated approach in a different antigen context.	NCT06051695
**Sequential AND-Gate CAR-T** *SynNotch “IF/THEN” CAR (E-SYNC)*	Glioblastoma (GBM) (Priming: EGFRvIII → Effector: EphA2/IL-13Rα2)	Phase 1	First-in-human trial. Recently initiated (2024). Will assess safety, feasibility, and optimal dosing of intravenously administered SynNotch CAR-T cells for GBM. Represents a clinical test of complex synthetic biology circuitry.	NCT06186401
**Inducible Suicide Switch** *iCasp9 safety switch*	Various (often used as an add-on safety feature to CD19, BCMA, or other CAR-T constructs)	Multiple Phase 1/2 trials (e.g., for AML, ALL, lymphoma)	Administration of the dimerizing drug (AP1903/rimiducid) leads to rapid elimination of >90% of CAR-T cells within 30–60 minutes, effectively managing severe toxicities like CRS or ICANS. Now a benchmark safety component in many next-gen CAR-T designs.	(69)
**Drug-Controlled ON-Switch** *Rapamycin/Dimerizer-based CARs*	Various (Preclinical target: CD19, others)	Early Phase 1 (limited published data)	Robust preclinical models, clinical translation faces challenges related to the pharmacokinetics of the dimerizer drug and potential immunosuppressive side effects of molecules like rapamycin. Demonstrates the practical hurdles of pharmacologic control.	(70, 71)
**Adaptor-Mediated “Universal” CAR-T** *UniCAR, SUPRACAR, or Anti-FITC CAR platforms*	Various (e.g., PSMA, CD33, Tumor-associated antigens)	Early Phase 1 (e.g., UniCAR-T-CD123 in AML)	Early trials explore feasibility. Aim to demonstrate that soluble adapter infusion can redirect pre-made CAR-T cells to specific tumors, offering flexible, tunable targeting. Key challenges include immunogenicity of adapters and precise dosing kinetics.	(72, 73)

Beyond these dedicated logic-gated trials, safety switches like iCasp9 have become a clinical mainstay, successfully integrated into numerous CAR-T products to provide a reliable emergency stop. However, the path to clinic for other elegant control strategies, such as small-molecule ON-switches or fully adaptable universal CAR-T systems, has been slower, highlighting the translational gaps between mechanistic proof-of-concept and reproducible, manufacturable, and pharmacologically sound human therapies (69).

Future clinical progress will depend on overcoming several key challenges (1): identifying robust tumor-specific antigen pairs or microenvironmental cues that hold across patient heterogeneity (2); optimizing the pharmacokinetics and immunogenicity of switch-inducing drugs or adapter molecules; and (3) developing companion diagnostics to select patients whose tumors express the required logic inputs. As these hurdles are addressed, the clinical pipeline for smart CAR-T therapies is poised to expand, offering hope for safer and more effective immunotherapy against a broader range of cancers.

### 3.1 Cytokine engineering for enhanced CAR-T function

Genetic co-expression of cytokines, creating so-called “armored” CAR-T cells, has emerged as a primary strategy to overcome the immunosuppressive tumor microenvironment and enhance persistence (75). For instance, CAR-T cells engineered to constitutively secrete IL-12 or IL-18 have demonstrated superior antitumor activity in solid tumor models by reprogramming the myeloid compartment, reducing suppressive cell populations, and promoting a pro-inflammatory milieu that supports T cell function (76, 77). A pivotal approach involves the inducible expression of cytokines under the control of synthetic receptors like synNotch, which restricts cytokine release to the tumor site. A landmark study showed that synNotch-driven IL-12 production by CAR-T cells triggered a powerful, localized immune response, leading to complete tumor regression in mouse models of pancreatic cancer and melanoma, while systemic toxicity was minimized due to the spatial control of cytokine release. Similarly, co-expression of IL-7 and CCL19 has been shown to promote CAR-T cell survival and recruitment into solid tumors, acting as a self-sustaining chemotactic and proliferative signal (78). These studies underscore the principle that engineering CAR-T cells to be autonomous producers of supportive cytokines can markedly improve their expansion, infiltration, and durability.

**Figure 5:**
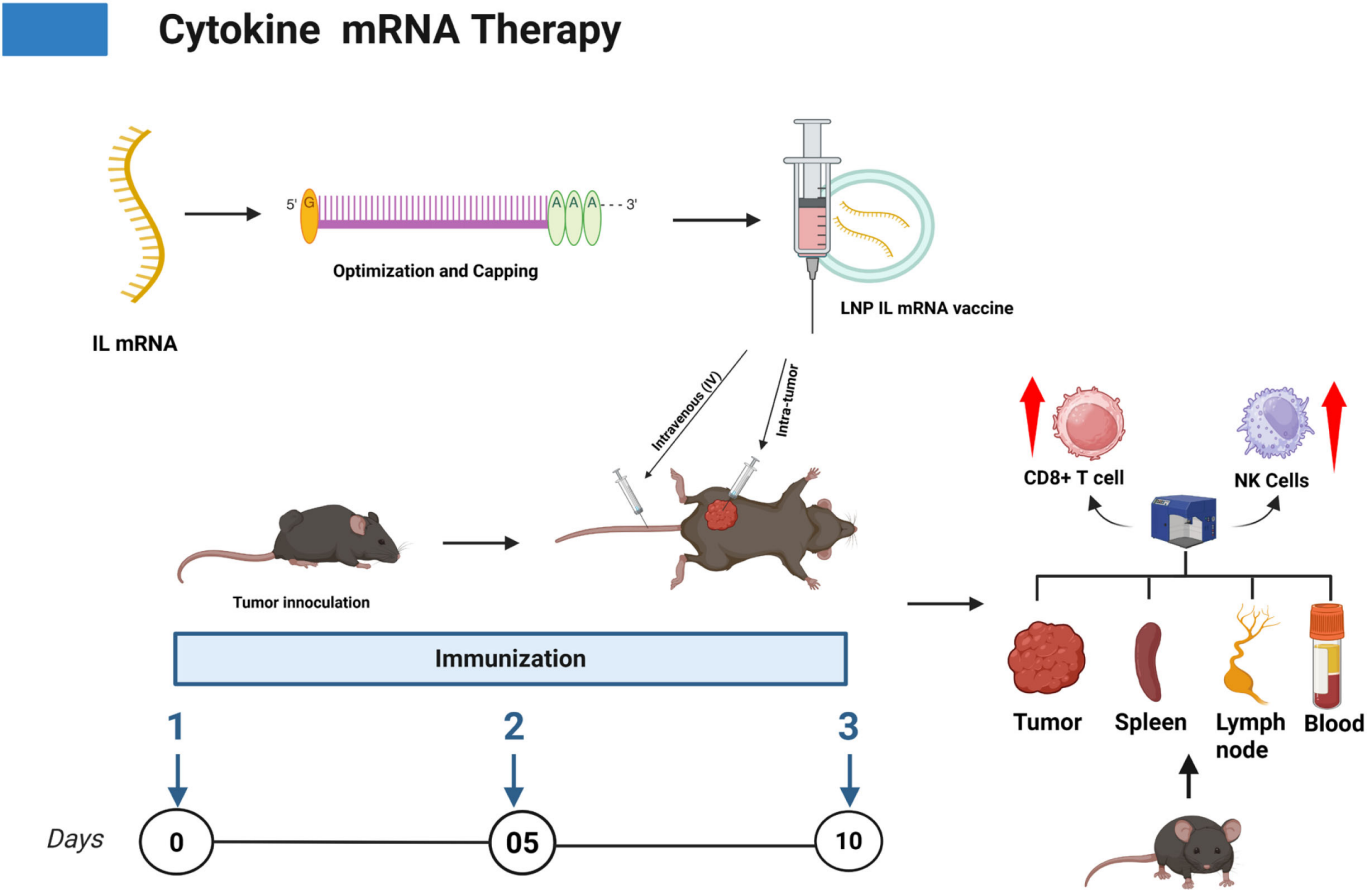
Shows the cytokine therapy (IL) for enhancing the NK cells and T cells in mice tumor.

Beyond genetic engineering, cytokine activity can be tethered to CAR-T cells through bioconjugation or delivered locally via biomaterial scaffolds. For example, CAR-T cells have been chemically conjugated with IL-15 super agonist complexes, providing a potent, membrane-bound survival signal that significantly enhances *in vivo* persistence and antitumor efficacy against leukemia and solid tumors without inducing systemic cytokine toxicity (79). Biomaterial platforms offer another dimension of control; injectable hydrogels co-delivering CAR-T cells and sustained-release cytokine depots (e.g., IL-15, IL-2) create a local immune niche that supports prolonged CAR-T cell activity and reduces off-tumor effects (79, 80). Clinically, these strategies are beginning to translate. Early-phase trials of armored CAR-T cells co-expressing cytokines like IL-12 or dominant-negative TGFβ receptors are underway for solid tumors (e.g., NCT03932565, NCT04976218), aiming to counteract TME suppression (77). Collectively, cytokine engineering, whether through genetic, chemical, or biomaterial means, represents a critical tool to endow CAR-T cells with the resilience and functionality required to succeed in the challenging landscape of solid cancers (**Figure 5**).

## 4 Comparative synthesis, challenges, and future perspectives

The rapid proliferation of “smart” CAR-T control strategies presents a diverse toolkit for overcoming the limitations of conventional therapy. However, their translational promise is counterbalanced by distinct and often significant practical hurdles. This section synthesizes the core attributes, limitations, and developmental stages of the major platforms (**Table 3**) and discusses overarching challenges and future directions for the field.

**Table 3:** Comparative analysis of major smart control strategies for CAR-T cell therapy.

Control strategy	Primary mechanism	Key benefits	Major limitations & translational challenges	Stage of development	Tumor application
Light-Based (Optogenetic) Control (e.g., LiCAR, UCNP-LiCAR)	External physical trigger (light) induces protein dimerization or gene expression.	Unparalleled spatiotemporal precision; rapidly reversible; tunable intensity.	Limited tissue penetration; requires specialized hardware; immunogenicity risk of exogenous photoreceptors.	Preclinical (advanced *in vivo* proof-of-concept).	Solid Tumors (superficial or accessible via devices).
Pharmacologic Molecular Switches (e.g., ON-Switch, iCasp9, UniCAR)	Small-molecule drug induces CAR assembly, apoptosis (iCasp9), inhibition, or bridges antigen recognition.	Tunable and reversible action; clinically validated safety switch (iCasp9); flexible antigen targeting.	Drug PK/PD challenges (sustained levels needed); potential off-target drug effects; complex dosing regimens.	Mixed: iCasp9 is clinically validated; ON-switches are in Early Phase I; Dasatinib is repurposed.	Broad (Hematological & Solid).
Biomaterial Platforms (e.g., Hydrogels, Scaffolds)	Bioactive matrix localizes, protects, and provides stimulatory signals to CAR-T cells *in situ*.	Localizes activity to tumor site; enhances persistence in hostile TME; enables *in vivo* manufacturing.	Invasive delivery required; limited to localized disease; biocompatibility and regulatory complexity.	Preclinical to early clinical translation.	Solid Tumors (localized, resectable, or injectable).
Synthetic Logic Circuits (e.g., AND/NOT Gates, SynNotch)	Boolean logic (AND, NOT, IF-THEN) processes multiple antigen inputs to control activation.	Fundamentally enhances specificity; reduces on-target/off-tumor toxicity; tackles antigen heterogeneity.	High genetic payload complexity; risk of circuit malfunction; difficult GMP manufacturing for multi-gene constructs.	Early Clinical: Tmod and SynNotch (Phase I/II). Preclinical: Complex multi-gate circuits.	Solid Tumors (where specific antigen combinations can be defined).
Cytokine Engineering (e.g., Armored CARs IL-12/15)	Genetic co-expression, tethering, or localized release of immunomodulatory cytokines.	Counteracts immunosuppressive TME; enhances CAR-T expansion, persistence, and metabolic fitness.	Risk of exacerbating toxicity (e.g., CRS); potential systemic effects if not controlled; risk of T-cell exhaustion.	Early Clinical: Armored CARs (Phase I/II). Preclinical: Advanced delivery systems.	Solid Tumors (primary focus to overcome TME barriers).
Vaccine-Boosted CAR-T (e.g., mRNA, Viral Vector, DC-based)	Engages endogenous immunity (DCs, T cells) via co-stimulation for broad antitumor response.	Induces epitope spreading; combats antigen escape; generates durable immune memory.			

### 4.1 Integrated analysis and cross-cutting challenges

The comparative analysis presented in **Table 3** underscores several pervasive and interdependent challenges that delineate the current translational frontier of smart CAR-T cell development. First, a fundamental trade-off exists between specificity and simplicity. Platforms engineered for maximal discriminatory precision, such as multi-antigen logic gates or patient-specific neoantigen vaccines, incur substantial costs in genetic payload complexity, manufacturing sophistication, and the prerequisite for comprehensive biomarker-based patient stratification (81). Conversely, more generalized control modalities, including systemic pharmacologic switches or constitutive cytokine secretion, frequently struggle to attain the tumor-restricted selectivity necessary to mitigate on-target, off-tumor toxicity in solid malignancies (82). Second, a significant clinical delivery barrier impedes many high-precision strategies. Optogenetic systems and certain biomaterial platforms are contingent upon sophisticated, and often invasive, enabling technologies, including specialized illumination hardware, implantable devices, or chronic intravenous infusions of adapter molecules (83). Their clinical realization thus mandates concurrent advancements in bioengineering and medical device integration. Third, the risk of immunogenicity presents a persistent translational obstacle. The incorporation of xenogeneic protein domains (e.g., microbial photoreceptors, bacterial enzymes in suicide switches) or novel synthetic epitopes (e.g., adapter-recognition tags) can elicit host adaptive immune responses, potentially leading to the premature elimination of engineered cells and compromising both the durability and safety of the therapy. Finally, these platforms face formidable manufacturing and regulatory complexities. The sequential integration of multiple functional genetic modules into a single living therapeutic exponentially amplifies the challenges associated with Good Manufacturing Practice (GMP) production, batch-to-batch quality control, and regulatory approval. Establishing definitive potency and release assays for a cell product equipped with layered decision-making circuitry is intrinsically more complex than for a conventional single-antigen targeted CAR-T construct.

### 4.2 Future perspectives: convergence and personalization

Future progress in smart CAR-T cell therapy is unlikely to be driven by the dominance of any singular technological approach. Instead, the field is poised to evolve through the intelligent convergence of multiple strategies within a comprehensive precision oncology framework. The next therapeutic generation may feature combinatorial platforms where biomaterial scaffolds locally deliver cytokine-enhanced, logic-gated CAR-T cells, whose activity is further modulated via pharmacologic switches and consolidated through concomitant administration of personalized mRNA vaccines (84). This multi-modal integration would concurrently tackle the challenges of tumor localization, immunosuppressive microenvironment remodeling, target specificity, safety control, and long-term immunological memory (85). Furthermore, advances in synthetic biology promise a shift from static, pre-programmed circuits toward dynamic, closed-loop systems. Next-generation CAR-T cells could be engineered with biosensors to detect tumor microenvironmental cues, such as metabolic perturbations, checkpoint ligand density, or intrinsic activation states, enabling autonomous phenotypic adaptation, including cytokine secretion modulation or memory differentiation (86). The implementation of such sophisticated designs will be underpinned by artificial intelligence, which will be instrumental in optimizing synthetic gene circuits, predicting immunogenic neoantigens for vaccine design, and, critically, interpreting patient multi-omics data to guide the selection of personalized therapeutic logic. However, for these innovations to achieve broad clinical impact, streamlined translation pathways are essential. Demonstrating scalable, cost-effective manufacturing and establishing manageable clinical workflows are prerequisites for widespread adoption, necessitating a deliberate balance between biological complexity and practical translatability. In summary, the transition from broadly active cellular agents to precision-engineered “living therapeutics” is actively unfolding. A critical appraisal of the comparative strengths and limitations of existing control paradigms provides a strategic roadmap for this evolution. The ultimate objective is the development of a new class of adaptive cellular immunotherapies capable of dynamically responding to the unique pathophysiological landscape of each individual malignancy.

## 5 Conclusion

The evolution of CAR-T cell therapy from a static, constitutively active agent into a dynamically programmable “living drug” represents a pivotal paradigm shift in cancer immunotherapy. This review has delineated the rapidly expanding arsenal of smart control strategies—spanning optogenetic triggers, pharmacologic switches, biomaterial scaffolds, synthetic logic circuits, cytokine engineering, and vaccine-boosting approaches, each designed to impose precision, safety, and adaptability upon T cell effector functions. First-generation CAR-T therapies have demonstrated transformative success in hematologic malignancies, their broader application, particularly against solid tumors, has been constrained by on-target/off-tumor toxicity, antigen escape, immunosuppressive microenvironments, and a lack of temporal control. The technologies surveyed herein directly confront these limitations, offering mechanisms to spatially localize activity, titrate effector responses, implement Boolean antigen discrimination, and reprogram the tumor niche.

However, the path to clinical translation is not defined by the supremacy of any single strategy but by a critical understanding of their inherent trade-offs. As our comparative analysis reveals, a fundamental tension exists between specificity and simplicity: the most discriminating systems (e.g., multi-input logic gates, personalized vaccines) incur substantial costs in genetic complexity, manufacturing rigor, and patient stratification, whereas simpler, systemically applied controls (e.g., pharmacologic switches) may lack sufficient tumor selectivity. Furthermore, significant translational barriers persist, including the clinical delivery hurdles of invasive enabling technologies, the persistent risk of immunogenicity from foreign protein domains, and the daunting manufacturing and regulatory complexities of multi-module living therapeutics.

Looking forward, the next frontier lies not in the isolated optimization of these platforms, but in their intelligent convergence within a precision medicine framework. Future iterations may combine biomaterial-based local delivery of cytokine-armed, logic-gated CAR-T cells, whose activity is further fine-tuned via pharmacologic switches and consolidated through concomitant neoantigen vaccination. Advances in synthetic biology will pave the way for closed-loop, autonomous systems capable of sensing and dynamically responding to microenvironmental cues—such as metabolite levels, checkpoint density, or cellular activation states, to self-regulate phenotype and function. The realization of such sophisticated designs will be accelerated by artificial intelligence, which will be indispensable for optimizing genetic circuits, predicting actionable neoantigens, and, most critically, interpreting patient multi-omics data to guide the selection of personalized therapeutic logic.

Ultimately, for these innovations to transition from compelling proof-of-concept to widespread clinical impact, the field must diligently address the imperatives of scalable manufacturing, cost-effective production, and streamlined clinical workflows. The journey from blunt cellular instruments to precision surgical tools in immunotherapy is well underway. By navigating the intricate landscape of smart control strategies with a clear-eyed assessment of both their formidable potential and their associated challenges, researchers and clinicians can strategically engineer a new generation of adaptive cellular therapeutics. These next-generation agents will be precisely calibrated to navigate the unique biological terrain of each patient’s malignancy, finally realizing the promise of safe, effective, and durable immunotherapy for all cancers.
